# Medical Science in the Queen's Reign

**Published:** 1897-06-19

**Authors:** 


Vol. XXII.—No. 560.] [June 19, 1897.

### Medical Science in the Queen's Reign.

### The Diamond Jubilee.

The coming week is likely to be marked by such a demonstration of affection and respect for the reigning Sovereign as would, perhaps, be impossible in any other country.

That Her Majesty should have reached the age of seventy-eight, that she should at that age retain good health, and should remain in the full possession of all her faculties is matter for much congratulation, and is full excuse for all those jubilant congratulations which will shortly rise from every corner of the land. Nevertheless we can quite believe that people will be found who will say that it is nothing wonderful, and that as for the fact that this reign has lasted longer than any other, it is a mere accident. What is wonderful, however, is the celebration itself, the unanimous loyalty of the whole nation, the fact that in an era the very keynote of which has been change, the casting off of all that is old, the search for novelty, and the willingness displayed on every side to throw down old gods and to set up new, the throne should, in such increasing degree, have maintained its hold upon the people, and that amid so many crumbling faiths affection for the Queen should have become so universal and deeply fixed a sentiment among her subjects as to lead to such a demonstration of loyalty and respect as that on the eve of which we write. What is the cause? The cause we take it is to be expressed in the one word—sympathy.

### The Bond of Union.

Ever since the Queen ascended the throne it has always been apparent to her people that when they suffered she suffered, that what interested them interested her also. But sympathy cannot be, and has not been, one-sided. The pleasures of our Queen's life, so far as they have been known to the people, have been domestic pleasures, pleasures into which all her people could enter, and with which they could sympathise; and the troubles by which Her Majesty has been known to be afflicted have been exactly of that kind which sooner or later come upon every family, and by these troubles, perhaps, more than by anything else has the bond of union between the Queen and her people been cemented.

### The Prince Consort.

It will not do in days of jubilee to forget the dead, and even while offering congratulations to Her Majesty we ought never to forget how much we owe to one who is no longer here, to Albert, Prince Consort, who in the short twenty-one years that he lived among us, exercised an influence for good which has continued ever since.

In looking back then to the progress made during the past sixty years we must always remember the patronage bestowed, and the social recognition given, by Prince Albert to the arts and sciences at a time when science, at any rate, did not stand high in the consideration of the world.

### Medicine as a Science.

This brings us to the point which, after all, is the important one, that it is as a science that medicine has made such strides during the Queen's reign. It was want of knowledge that caused medicine at the beginning of the reign to be still a mere art and surgery a mere handicraft, as on the other hand it is not because the present race of physicians and surgeons are better, or cleverer, or more devoted to their work than those of old, but because they approach their work in a scientific spirit that they are able to obtain the success they do at the present day.

### The Birth of Bacteriology.

It is a curious and an interesting thing to note that in the year in which the Queen ascended the throne the foundation of what we now know in regard to septic processes, and in reality the very germ of the antiseptic treatment, was being laid by scientists who were at that time beginning to associate the growth of micro-organisms with decomposition and disease.

It was in 1837 that Donné described the infusorian which he supposed was concerned in the infection of syphilis, and although he had ultimately to give up that idea, still the dead hypothesis left as a legacy the suggestion, which has been so fertile in the hands of others, of the existence of a *contagium vivum*, a living germ, by the growth of which disease is both produced and spread. In the same year Schwann and Cagniard Latour announced the results of their investigations into the relations between the growth of the yeast-plant and the occurrence of fermentation, and again in the same year Bassi described the minute spores or growths in the bodies of the silkworms which were affected with silkworm disease, which were afterwards shown by Pasteur to be the cause of the malady which was causing such loss to the breeders of these worms.

### The Differentiation of the Microbes.

By degrees it became clear that while yeasts gave rise to fermentations, the product of this fermentation was not always the same, more than one ferment was involved, and the process sometimes ended in the production of alcohol, sometimes of lactic acid.

At the same time it was found that bacteria, in many cases, gave rise to decomposition, and then at last, but not till twelve years after the Queen's reign had commenced, it was discovered that bacteria were associated with splenic fever.

### The Discovery of the Cell.

To gain the slightest idea of the enormous changes which have taken place with the Queen's reign not merely in the amount of our knowledge, but in the very standpoint from which things are looked at, we must remember that although the cell had already been observed as the unit of the structure in plants, it is less than sixty years since Schwann published his observations establishing the cell theory in regard to animal tissues.

It is difficult indeed to imagine how people thought about histological and biological problems, and what sort of conceptions they can have had in regard to vital processes when they did not even recognise the existence of a cell as a vital unit. Not till the Queen had been twenty years upon the throne did Virchow's "Cellular-pathologie" appear. Needless to say that during the first half of the reign our knowledge of the pathology of morbid growths, as it is now understood, was an absolute blank.

### The Contagium Vivum.

But, meanwhile, a scientific investigation of the cause of infectious disease had been going on upon quite different lines. England had been twice ravaged by cholera, and the researches of Snow had shown that this disease was disseminated by water, and although his investigations did not specially aim at proving the living nature of the cholera infection, the only possible conclusion to be drawn from the facts that he placed on record was that infectious epidemic diseases, such as cholera, were due to specific organisms, to a *contagium vivum* acting on the tissues of the patients, much as the *torulæ* and the bacteria had been shown by the investigations of the microscopists to be the cause of fermentation and decomposition. Then came Pasteur, Lister, Burdon-Sanderson, Koch, Roux, and many others, whose names we delight to honour. But the fact on which we wish here to insist is that the whole of this knowledge—which is the scientific basis of so very large a part of our procedures in surgery, of our theories in medicine, and of our preventive measures in that totally new science, sanitation—has all arisen within the time that the Queen has sat upon the throne, and that the very beginning of it, the very first inkling of what is now part of the elementary science catechisms taught in schools was published to the world as an absolutely new thing in the very year of her Majesty's accession.

### Theories of Disease.

The popularisation of the leading idea which underlies antiseptic surgery and the great publicity which has been given to all that has had to do with the production of anti-toxins have undoubtedly led many people to look upon bacteriology as a matter interesting chiefly to operative surgeons, and to those who are concerned with the treatment of infectious disorders. It should, however, be constantly borne in mind how great an influence upon pathology in general has been exerted by the knowledge that many diseases are due to the growth of micro-organisms within the body. This idea, which practically came in with the reign, an idea the development and extension of which has been one of the most striking and all-pervading features of the science of the period, has had an extraordinary influence in modifying our views of the nature of many disorders. Diseases which used to be looked upon as entities to be attacked with violent remedies and driven out of the body, and at a later period were treated as mere perversions of normal function, are now recognised as being due in part to the evil effects of morbid products resulting from microbic growth, and in part to the reaction of the body—efforts to throw off the evil influences to which it is being subjected, attempts to accommodate itself to the new conditions in which it finds itself. In one sense, then, we see a revival of the old idea that disease is an entity to be assailed; we recognise again the truth of the phrase which describes people as being attacked by a disease, and we become conscious that the processes of many maladies are but indications of a struggle for the mastery. The conflict, however, is not now between the physician and the malady, but between the patient and microbe. The physician looks after the commissariat, takes care that other enemies do not intervene, sees to the removal of the débris of the fight, and sometimes is able to provide his patient with munitions of war, in the shape of anti-toxins, by which to beat off the assailants. Thus the physician may give assistance at every turn. But the actual battle must be fought out between man's tissues and the infection which attacks them.

### The Beginnings of Sanitation.

All our increasing knowledge points then to the importance of the tissues being maintained in full vitality, as they can only be when man's surroundings are conducive to health.

The recognition of this has led to the development of a new branch of medical science, viz., sanitation or public health, a branch which is entirely the growth of the present reign. To show how completely the history of sanitation is included within the last sixty years, it is well to recall the fact that it was in the very year of the Queen's accession that it for the first time became possible to obtain statistics even on such elementary matters as births and deaths; for it was but in 1837 that the Act for registering births, deaths, and marriages became law. Considering the complete information which we are now accustomed to receive as to the daily progress of any epidemic which may happen to occur in our midst, it is curious to recall that the actual extent of the fatality of the great epidemic of cholera which swept over England in 1831-32 was never known. It was estimated to be somewhere between 50,000 and 60,000, but in default of registration of deaths the exact number of the dead could not be ascertained.

### Water and Disease.

The water carriage of infection is now so well recognised a factor in the production of epidemics, and purity of water supply is so generally accepted as an essential to the maintenance of health, that it is worth remembering that the Queen had been on the throne for nearly seventeen years, and two epidemics of cholera had passed over the land, before Dr. Snow was able to demonstrate by aid of the celebrated Broad Street pump that even such a disease as cholera was waterborne. The condition of absolute ignorance in regard to the importance of pure water, and the callous indifference with which the whole subject was regarded for many years after the commencement of the reign may be judged of from the fact that for more than a dozen years after the Queen's accession none of the London water companies obtained their water from higher up the Thames than Vauxhall Bridge.

### State Medicine.

But if we take State medicine in its modern sense, it was not even dreamed of in the early days of the reign. Individualism was rampant. One's duty towards one's neighbour was comprised in the poor law, and in the regulations of the workhouse. Overcrowding, under-feeding, and absence of all sanitation was so much the order of the day that 2,000 cases of typhus were received annually into the London Fever Hospital—a disease which now has almost vanished from among us.

